# Fiber Bragg Grating Displacement Sensor with High Abrasion Resistance for a Steel Spring Floating Slab Damping Track

**DOI:** 10.3390/s18061899

**Published:** 2018-06-11

**Authors:** Yongxing Guo, Wenlong Liu, Li Xiong, Yi Kuang, Heng Wu, Honghai Liu

**Affiliations:** 1 Key Laboratory of Metallurgical Equipment and Control Technology, Ministry of Education, Wuhan University of Science and Technology, Wuhan 430081, China; lwlwjc@126.com (W.L.); xiongli166@163.com (L.X.); kuangyi1993@163.com (Y.K.); wuheng6@126.com (H.W.); 2 Hubei Key Laboratory of Mechanical Transmission and Manufacturing Engineering, Wuhan University of Science and Technology, Wuhan 430081, China; 3 State Key Laboratory of Mechanical System and Vibration, School of Mechanical Engineering, Shanghai Jiao Tong University, Shanghai 200240, China; honghai.liu@port.ac.uk

**Keywords:** Fiber Bragg grating (FBG), displacement sensor, floating slab track, abrasion resistance, structural health monitoring (SHM)

## Abstract

This paper presents a fiber Bragg grating (FBG) displacement sensor with high abrasion resistance for displacement monitoring of a steel spring floating slab damping track. A wedge-shaped sliding block and an equal-strength beam form a conversion mechanism to transfer displacement to the deflection of the beam, and the deflection-induced strain is exerted on two FBGs. A special linear guide rail-slider and a precision rolling bearing have been adopted onto the conversion mechanism, which turned sliding friction into rolling friction and thus significantly reduced the friction during frequent alternating displacement measuring. Sensing principle and the corresponding theoretical derivation have been demonstrated. Experiment results show that the sensor has a sensitivity of 34.32 pm/mm and a high resolution of 0.0029 mm within a measurement range of 0~90 mm. Besides, the sensor has also a good measurement capability for micro-displacement within a range of 0~3 mm. The repeatability error and hysteresis error are 1.416% and 0.323%, respectively. Good creep resistance and high abrasion resistance for alternating displacement measurement have also been presented by a performance test. These excellent performances satisfy the requirements of high precision and long-term stability in structural health monitoring for machinery equipment and civil engineering, especially in the displacement monitoring of a floating slab damping track.

## 1. Introduction

Displacement measurement is a basic and important subject in structural health monitoring (SHM). As far as we know, the research of displacement monitoring of floating slab damping track based on FBG sensors has not been reported yet. It is difficult to measure the steel spring floating slab damping track’s displacement of the subway or to monitor it in the long-term using the traditional electrical sensor, because of zero temperature drift, the mounted environment lacks sealing schedules, and difficulties of working in high voltage. Therefore, it is necessary to design a displacement sensor that can realize the long-term and real-time monitoring of the floating slab damping track under the effect of frequent alternating displacement. Various displacement sensors based on electronic or fiber-optic techniques have been developed and used in SHM. It’s well known that electromagnetic principle-based displacement sensors need to work with a power supply and are susceptible to electromagnetic interference. Besides, they are also prone to tedious wires jointing and zero temperature drift. In recent years, as a particular class of optical fiber sensors, fiber Bragg grating (FBG) have widely attracted attention because of their inherent advantages, such as immunity to electromagnetic interference and optical power fluctuations, small profile, light weight, no zero-temperature drift and the fact that multiple FBGs can be arrayed along a single fiber [1,2]. During the past few decades, FBG sensors have been widely used in mechanical equipment, civil engineering, bridge scour monitoring, aircraft, and robot [3,4,5,6,7,8], and have successfully obtained the measurement of many physical quantities such as displacement, vibration, force and strain [9,10,11,12,13].

For the FBG displacement sensor, in order to measure the external displacement, a conversion mechanism must be designed to convert the displacement information into an axial strain exerted on FBG. Traditionally, most of the conversion mechanisms are designed as spring [14], ring-type [15,16], cantilever beam and its evolution type [17,18,19], and other composite structures [20,21,22]. However, these structure designs have the weaknesses of low measurement accuracy due to friction losses and poor durability, especially when the sensor works under frequent alternating displacement brought by the measured object.

In recent years, some different packing and structure designs of FBG displacement sensor have been reported. For instance, Zou et al. connected a spring and FBG in series and achieved high sensitivity measuring [23]. However, in this design, the elasticity coefficient of spring is liable to change, which could affect the measurement accuracy in the long-term monitoring. What’s more, the practical application of the sensor is also limited by a small range. Tao et al. proposed an FBG displacement sensor based on a thin-wall ring [24,25]. Although the designed structure is compact and the temperature cross-sensitivity is also solved, it suffered from defects, such as low repeatability caused by inconsistency from the manually pasting process, and low resolution based on reflection spectrum bandwidth demodulation due to the use of an optical spectrum analyzer (OSA). Some different packaging designs have been also reported which can be designed into a temperature-insensitive displacement sensor based on cantilever beam structure and its evolution type [26,27,28], whose common characteristic is to detect the reflection spectrum broadening of FBG. As far as we know, the profile of the broadened spectrum of FBG is usually irregular due to the nonuniform strain on the grating, which may cause inaccurate experimental results. Although these designs can solve the problem of temperature cross-sensitivity, a professional spectrometer is usually needed for the monitoring of bandwidth. Furthermore, the advantage of the spectrometer is inferior to that of an FBG wavelength interrogator in terms of scanning frequency and cost, which is not conducive to long-term and real-time monitoring in practical engineering. Dong et al. [29] obliquely glued a single FBG onto the lateral side of the cantilever beam. However, it is difficult to guarantee that the FBG’s midpoint coincides exactly with the zero-strain layer of beam, which will result in the FBG reflected spectrum fluctuations, thus it is difficult to obtain precisely reflected spectrum bandwidth variations. Furthermore, there is also the defect that a photo-detector is needed for compensating the optical power fluctuation and inconvenient in multiplexing due to the utilize of the power demodulation method.

To solve these issues and limitations, various structure designs and demodulation methods have been proposed by researchers to improve the performance of FBG sensors to meet the needs of practical engineering. Compared with the packing and structure designs of FBG as mentioned above, researchers provided some different structure designs based on the reflected wavelength demodulation. For instance, Li et al. designed two high-sensitivity FBG displacement sensors [30,31], a single FBG is pre-tensioned and the two side points are fixed, efficiently avoiding the unwanted chirp effect of grating. However, the wedge-shaped sliding block and T-shaped cantilever beam convert the horizontal displacement into vertical displacement by sliding rather than rolling, which may result in the poor abrasion performance in reciprocating displacement measurement in long-term. As mentioned previously, although the problems of low sensitivity and cross-sensitivity have been solved, most of those displacement sensors are limited by small measuring ranges and poor durability. Furthermore, the measurement accuracy can’t be guaranteed due to friction losses when sensors are used for long-term monitoring under the effect of frequent alternating displacement and a harsh environment in the field of SHM.

In this paper, an improved FBG displacement sensor is proposed to improve the accuracy and abrasion resistance of the displacement measurement in the application of long-term monitoring of floating slab damping track. This improved structure design overcomes the defects such as the changeability of the elasticity coefficient of force-transmitting medium, and directional movement of sliding block which could bring negative influences on the measuring accuracy in the exiting design of FBG displacement sensors. This knowledge has been validated in our previous work [32]. Furthermore, compared with the displacement sensor in literature [32], a linear guide rail has been used instead of the sliding surface of the wedge-shaped sliding block, and the free end of the beam adopts a precision rolling bearing contacted with the top surface of the slider. Moreover, the packing method of these two gratings are all pasted instead of a grating being pasted while the other is bare grating. This configuration improves problems such as irregular errors due to the different packaging methods of gratings, and poor durability due to frictional losses under the effect of frequent alternating displacement. The sensing principle of the designed mechanical structure have been derived, and then the sensor prototype has been manufactured, installed and fully tested.

## 2. Principle and Structure Design

### 2.1. Mechanical Structure Design

**Figure 1:**
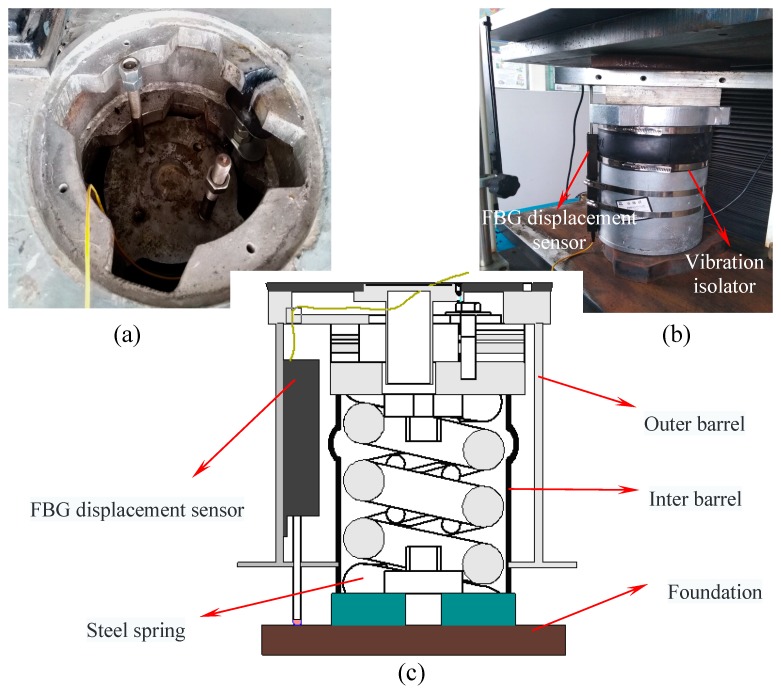
Installation situation of FBG displacement sensor: (**a**) Photo of field installation; (**b**) Installation testing; (**c**) Schematic diagram of installation.

In a subway line of Beijing, the dynamic impact by a passing high-speed train causes high stress in the rails and floating slab, thereby causing the alternating and creeping displacement. However, the allowable settlement range of the steel spring floating slab damping track is 0~3 mm. In order to guarantee the safe operation of the high-speed train, the quantitative value of rail alternating and creeping displacement must be monitored. All the sensors must be installed symmetrically in series along continuous floating slab damping track to form FBG sensing network for long-term and real-time displacement monitoring. All wavelength signal can be transmitted to an FBG interrogator by fiber optical cables and transmitted by the wavelength division multiplexing (WDM) technology. As shown in Figure 1, the FBG displacement sensor that meets design requirements is installed in between the outer barrel and inter barrel of vibration isolator.

**Figure 2:**
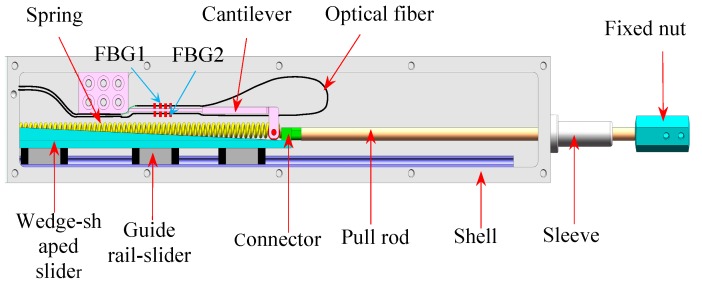
Structure of the proposed sensor.

**Figure 3:**
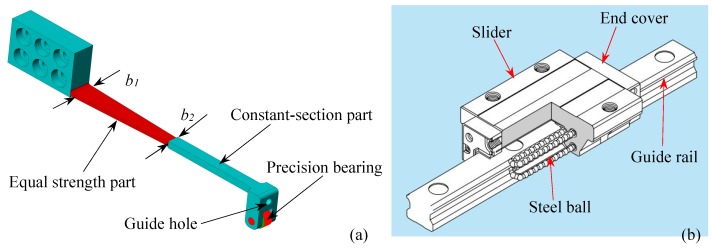
The detailed 3D diagram of designed structure: (**a**) Cantilever beam with a precision bearing; (**b**) Linear guide rail components.

In view of the requirements for high accuracy and high abrasion resistance of long-term monitoring of the floating slab damping track under the effect of frequent alternating displacement, this paper introduces our recent work on the design and investigation of a new FBG displacement sensor with high abrasion resistance. The mechanical structure design of the proposed sensor is shown in Figure 2. This sensor mainly consists of two FBGs with different wavelengths, a variable section cantilever beam, a wedge-shaped sliding block, a linear guide rail-slider, a pull rod and a restoring spring. Two FBGs in a single mode optical fiber are adhered on the upper and lower surfaces of the cantilever beam. The packing method of FBG can achieve differential measuring and solve the problem of temperature cross-sensitivity. Besides, in order to overcome the shortcoming of inaccurate measurement due to the frictional losses of mechanical conversion structures, the design of sensing structure has been improved. The detailed 3D diagram of cantilever beam and linear guide rail components are shown in Figure 3. A precision bearing has been adopted on the free end of the cantilever to contact with the surface of wedge-shaped slider. A special linear guide rail-slider has been used as a medium to fix the wedge-shaped slider to the base. These designs make all the sliding friction in our previous work [24] turn into rolling friction, which considerably reduces the friction. A special connector is designed to fix one end of the spring and the pull rod on the wedge-shaped slider. The pull rod receives the external displacement inputs and then drives the wedge-shaped slider. Meanwhile, the spring can effectively ensure the pull rod has a good reciprocating ability to move with the measured object. Therefore, the external displacement can be converted into the flexural strain of the cantilever beam, and the displacement-induced strain can be exerted on the two FBGs. So the displacement is determined by the structural parameters of the conversion mechanism. The corresponding theoretical derivation will be presented as follows.

### 2.2. Measuring Principle

The reflected wavelength shift of FBG is influenced by the changes of axial strain and environment temperature. The relation between center reflective wavelength shifts and strain/temperature can be described by:

(1) $$\frac{\Delta \lambda }{\lambda }=\left(1-P_{e}\right)\Delta \varepsilon +\left(\alpha_{f}+\xi \right)\Delta T$$

where Δλ is the center wavelength shift of FBG, *λ* is the initial wavelength of FBG, $\alpha_{f}$ is the thermal expansion coefficient, ξ is the thermal-optic coefficient and $P_{e}$(≈0.22 at room temperature) is the effective photo-elastic coefficient.

**Figure 4:**
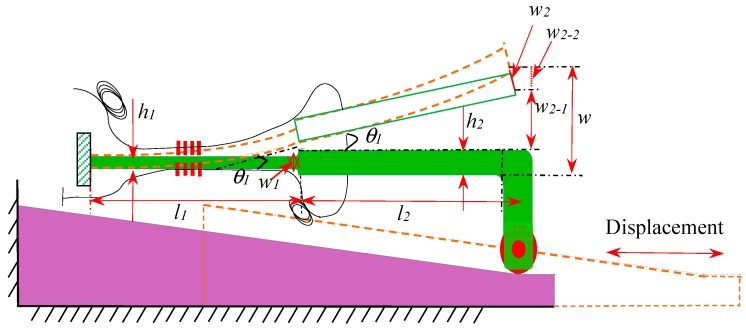
Schematic diagram for the sensing principle of the proposed sensor.

As shown in Figure 2a, the cantilever beam includes an equal-strength part and a constant-section part. The thickness of the constant-section part is larger than the equal-strength part where the FBGs are glued, which could bring higher strain detection sensitivity for FBG because the bending strain concentrates on the thinner equal-strength part. Figure 4 shows the schematic diagram of the conversion mechanism. FBG1 and FBG2 are glued on the upper and lower surfaces of equal-strength part, respectively. Using the difference of the wavelength shifts brought by the two opposite surface strains as the sensing signal, the temperature cross-sensitivity could be avoided, and the sensitivity can be improved. The relationship between the measured displacement S and the output of wavelength shifts difference $\Delta \lambda_{2-1}$ is deduced below.

As shown in Figure 4, it can be seen that the height difference of the wedge-shaped slider is the total deflection W. What’s more, the cantilever beam includes two parts, whereas these two FBGs are glued on the equal-strength part. Therefore, it is necessary to obtain the theoretical relationship between the deflection w_1_ of the equal-strength part and the total deflection W. Meanwhile, we can see that vertical displacement of the constant-section part contains two parts: the displacement ($w_{2-1}$) because of the rotation angle $\theta_{1}$ and the component of $w_{2}$ along the vertical direction ($w_{2-2}$). $w_{2-1}$ equals $L_{2}sin\theta_{1}$, and $w_{2-2}$ equals $w_{2}cos\theta_{1}$. So $W=w_{1}+w_{2-1}+w_{2-2}$.

According to the transfer principle of the deflection for the cantilever, one can obtain the following expression:

(2) $$w_{1}=\;W-L_{2}sin\theta_{1}-\;w_{2}cos\theta_{1}$$

where $L_{2}$ is the length of the constant-section part.

According to material mechanics, the equal force F is exerted on the equal-strength part and constant-section part, the deflection $w_{1}$ of the equal-strength cantilever can be expressed as:

(3) $$w_{1}=\frac{F{L_{1}}^{3}}{EI}=\frac{6FL_{1}}{Eb_{1}{h_{1}}^{3}},$$

where $b_{1}$, $h_{1}$, $L_{1}$ is the width, thickness, and length of the equal-strength part, respectively; E, I is Young’s modulus and the section moment of inertia, respectively.

According to material mechanics, the equations of rotation angle and moment of inertia can be expressed as:

(4) $$\theta_{x}=\frac{dw}{dx}=\int \frac{F(L_{1}-x)}{EI_{x}}dx+C$$

(5) $$I_{x}=\frac{h^{3}}{12}\left(\frac{L_{1}-x}{L_{1}}\right)b_{1}.$$

where x is the distance between equal-strength cantilever and the fixed end; $I_{x}$ is the corresponding moment of inertia at x.

Substituting Equation (5) in Equation (4) we can get:

(6) $$\theta_{x}=\frac{12FL_{1}}{Eb_{1}h^{3}}x+C.$$

Then, the rotation angle $\theta_{1}$ can be expressed as:

(7) $$\theta_{1}=\frac{12F{L_{1}}^{2}}{Eb_{1}{h_{1}}^{3}}.$$

According to material mechanics, the deflection $w_{2}$ of the equal-section cantilever can be expressed as:

(8) $$w_{2}=\frac{4{L_{2}}^{3}}{Eb_{2}{h_{2}}^{3}}\times F$$

where $b_{2}$, $h_{2}$ is the width and thickness of the equal-section cantilever, respectively; E is Young’s modulus of the cantilever beam.

Combining Equations (2), (3), (7) and (8), the total deflection W can be written as:

(9) $$W=\frac{6F{L_{1}}^{3}}{Eb_{1}{h_{1}}^{3}}+\sin\frac{12F{L_{1}}^{2}}{Eb_{1}{h_{1}}^{3}}\times L_{2}+\cos\frac{12F{L_{1}}^{2}}{Eb_{1}{h_{1}}^{3}}\times \frac{4F{L_{2}}^{3}}{Eb_{2}{h_{2}}^{3}}.$$

For the design of cantilever beam in this paper, $sin\theta_{1}$ is small, which can be approximated to $\theta_{1}$, and $cos\theta_{1}$ can be approximated to 1, so Equation (9) can be transformed to

(10) $$W=\frac{6F{L_{1}}^{3}}{Eb_{1}{h_{1}}^{3}}+\frac{12F{L_{1}}^{2}L_{2}}{Eb_{1}{h_{1}}^{3}}+\frac{4F{L_{2}}^{3}}{Eb_{2}{h_{2}}^{3}}.$$

Substituting the relevant parameters of designed cantilever such as $b_{1}=6mm$, $b_{2}=3mm$, $L_{1}=L_{2}=30mm$, $h_{1}=1mm$ and $h_{2}=2mm$ into Equation (10), the relationship between the deflection *w*_1_ and the total deflection W can be regarded as: $w_{1}=\left(6/19\right)W$. Then, combining the deflection *w*_1_ and the strain *ε* of the equal-strength beam, we can get the equation: $\varepsilon =h_{1}w_{1}/{L_{1}}^{2}$. Moreover, according to the sensing principle of this proposed sensor, the relationship of displacement *S*, total deflection *W* and dip rotation angle θ can be written as W=S×tanθ.

So we can get the theoretical relationship between the measured displacement S and the strain ε can be expressed as:

(11) $$S=\varepsilon \times \frac{19{L_{1}}^{2}}{6h_{1}\tan\theta }.$$

Based on the differential measurement method and the cantilever structure, the Bragg wavelength shifts of two FBGs are in opposite directions with the same absolute values. Moreover, these two FBGs are very close to each other, their temperature-induced shifts are considered to be identical. So the difference $\Delta \lambda_{2-1}$ of the two shifted Bragg wavelengths can be expressed as:

(12) $$\Delta \lambda_{2-1}=\Delta \lambda_{2}-\Delta \lambda_{1}=2\lambda (1-P_{e})\varepsilon$$

Combining Equations (11) and (12), the relationship of the wavelength shift difference and the measured displacement can be expressed as:

(13) $$\Delta \lambda_{2-1}=\lambda (1-P_{e})\frac{12Sh_{1}\tan\theta }{19{L_{1}}^{2}}$$

Equation (13) illustrates that the wavelength shift difference $\Delta \lambda_{2-1}$ of two FBGs is linearly related to the displacement S, and the external displacement can be effectively obtained by the center wavelength shift of FBG.

## 3. Sensor Prototype Manufacturing and Experiments

### 3.1. Sensor Prototype Manufacturing

**Figure 5:**
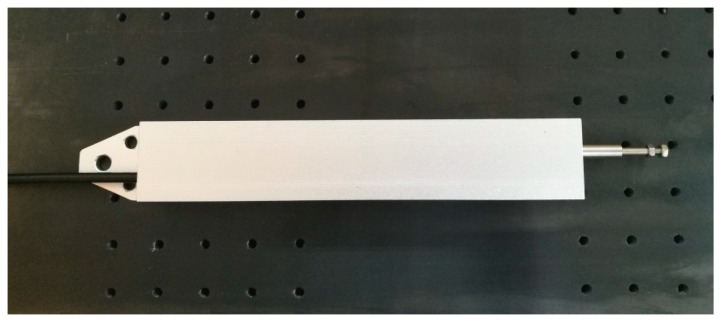
Photo of the FBG displacement sensor.

In addition to the parameters mentioned above, other parameters, such as the chute length in base, the height difference and length of wedge-shaped sliding block are regarded as 200 mm, 4 mm and 110 mm, respectively. So the measuring range of the proposed sensor can reach 90mm. Two FBGs with reflectivity of 90% and bandwidth of 0.18 nm have been glued on the cantilever beam by using a commercial adhesive (353ND, made by Epoxy Technology, Inc., Billerica, MA, USA). After assembling, a displacement sensor prototype with central wavelengths of 1530.1361 nm and 1533.2539 nm for FBG1and FBG2 has been manufactured as shown in Figure 5.

### 3.2. Experiments for Calibration and Test

**Figure 6:**
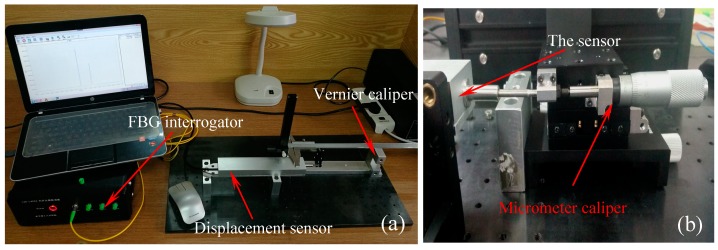
Photo of the experimental setup for displacement testing: (**a**) Large displacement measurement; (**b**) Micro-displacement measurement.

Figure 6 shows the photo of the experiment setup for displacement calibration test. A vernier caliper (accuracy: 0.02 mm), a micrometer caliper (accuracy: 0.01 mm) and the sensor prototype have been fixed on the experimental platform. As shown in Figure 6a,b, the vernier scale and micrometer caliper have been fixed together with the pull rod, respectively, and the pull rod can freely slide along the horizontal direction. A homemade FBG interrogator (sampling rate: 100 Hz, accuracy: 5 pm, resolution: 0.1 pm) is used to record the wavelength changes of the two FBGs under the external displacement.

**Figure 7:**
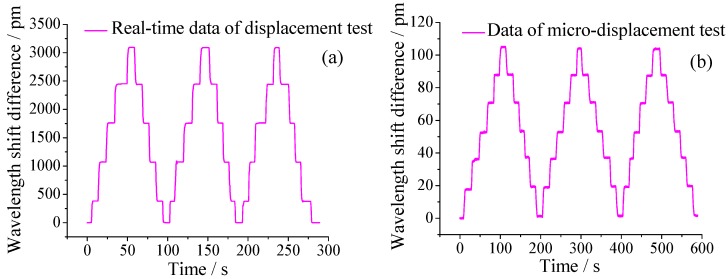
Time-history curve of three cycling tests: (**a**) Range of 0~90 mm; (**b**) Range of 0~3 mm.

The calibration experiment begins by stretching the pull rod from 0 mm to 10 mm, then stretching it to full-scale (90 mm) with a step of 20 mm and keeping 3–5 s for each displacement point, then unloading the displacement to 0 mm with the same step. As mentioned above, because the allowable settlement range of steel spring floating slab damping track is 0~3 mm, the capability of sensor to measure micro-displacement must be tested. The micro-displacement experiment begins by stretching it to 3 mm with a step of 0.5 mm and keeping 3–5 s for each displacement point. This loading and unloading test process has been repeated three times at a stable room temperature. The time-history wavelength changes of the three cycling tests of full-scale and micro-displacement have been plotted in Figure 7.

**Figure 8:**
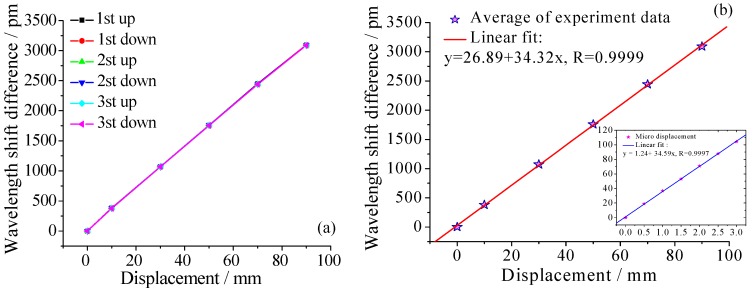
(**a**) The relationship of wavelength shift difference versus displacement; (**b**) Linear fit curve of average of experiment data and result of micro-displacement test.

Figure 8a shows the six curves of the relationship between different displacements and wavelength shift difference obtained from the time-history data. The results demonstrate that the variation patterns are linear and the repeatability error and hysteresis error for three cycling tests of the displacement sensor are 1.416% and 0.323%, respectively. Figure 8b shows variation of the average data of three repeated tests and the linear fitting curves which indicate good fits. From the fitted curve, the sensitivity of the sensor is observed to be 34.32 pm/mm with a linearity of 0.9999, and the measurement range is 0~90 mm. Besides, the thumbnails of Figure 8b is the fitted curve of the micro-displacement test, there is a little difference of the sensitivity between large-displacement and micro-displacement, it can be seen that the sensor has also a good measurement capability for micro-displacement. The wavelength resolution and accuracy of the used interrogator is 0.1 pm and 5 pm, consequently, this sensor can achieve a high resolution of 0.0029 mm and an accuracy of 0.15 mm.

**Figure 9:**
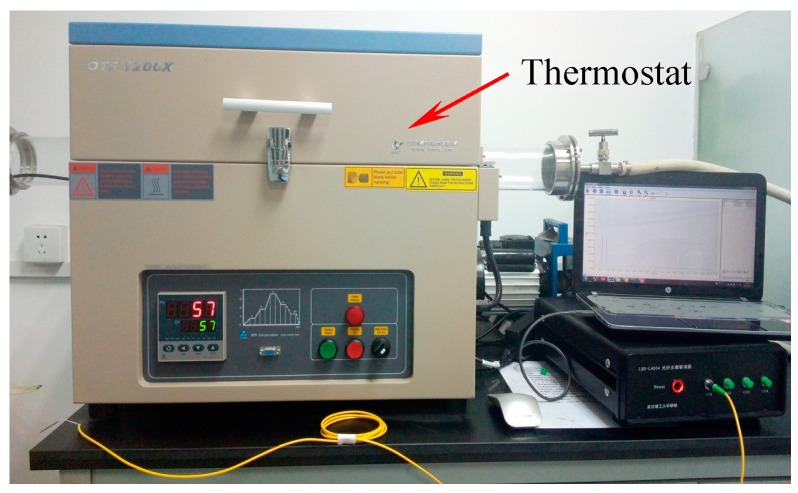
Photo of the experimental setup for temperature testing.

**Figure 10:**
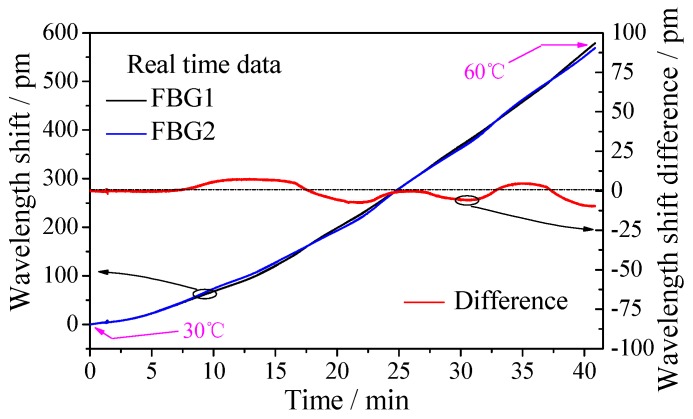
Time-history curve of temperature test.

Because the wavelength shift of FBG is influenced by both axial strain and temperature, knowledge of the temperature compensation ability of the displacement sensor is needed. As mentioned above, it can be seen that the temperature compensation method of the sensor is dual-grating difference output method with packaging full pasted FBGs. Compared with the reference grating method, the dual-grating difference output method reduces the big difference of the temperature sensitivity coefficient due to different packaging method of FBGs. As shown in Figure 9, a thermostat (OTF-1200X, HEFEI KE JING Materials Technology Co., Ltd. Hefei, China; accuracy: 1 °C, resolution: 0.1 °C) is used to change the surrounding temperature of the displacement sensor from 30 °C to 60 °C. Time-history curve of the two FBGs is shown in Figure 10. The maximum value of the wavelength shift difference in right Y axis is only 10 pm which indicates the sensor has good temperature compensation ability. The cause of the error is that the strain transfer ratio from FBG to substrate is affected by some random factors such as the length and thickness of adhesive area, consequently, which will affect the measurement accuracy of FBG.

As mentioned above, in the application of the floating slab damping track, the frequent alternating displacement brought about by external load often acts on the sensor in the long-term, which would affect the measurement accuracy due to the frictional losses of the mechanical structure in the sensor. What’s more, if the material performance of the elastic cantilever beam and the robustness of the bonding of FBG are affected by the frequent alternating displacement, the sensitivity of sensor also will change. Therefore, in order to make sensor have an applicability for this working condition of frequent alternating displacements, an abrasion resistance test for a sensor is indispensable.

**Figure 11:**
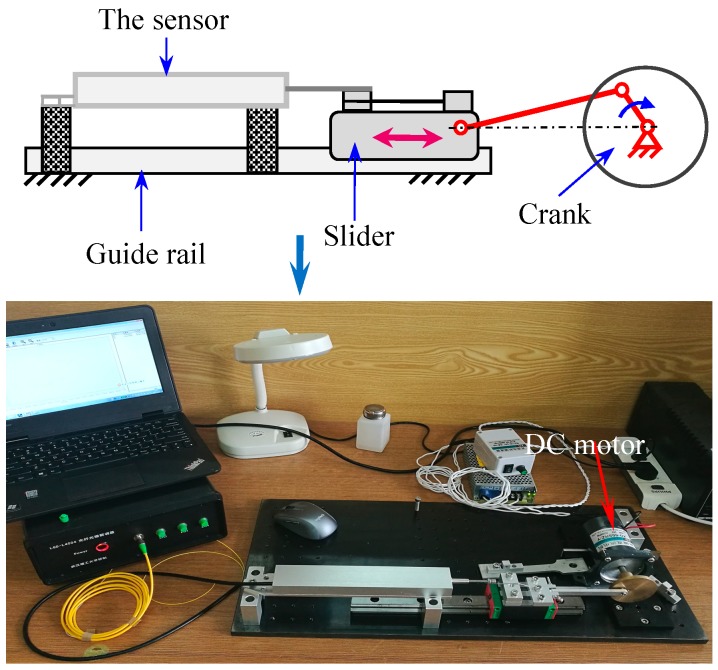
Principle and photo of the experimental equipment for alternating displacement test.

**Figure 12:**
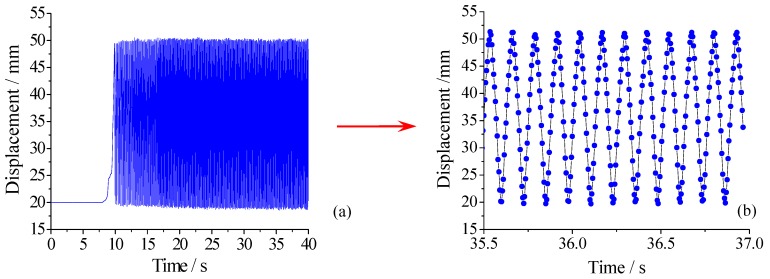
Part of the experimental data for alternating displacement test: (**a**) Real-time data of alternating displacement test; (**b**) The amplified data of 35.5 s~37 s.

As shown in Figure 11, special experimental equipment with the function of reciprocating motion has been designed using a crank-slider mechanism. The crank is powered by a DC motor and the pull rod of sensor is connected and fixed on the slider. Continuous alternating displacement from 20 to 52 mm has been applied on the sensor and the sensitivity and the measuring range of the sensor have been tested at different cycle numbers. Figure 12 shows part of the experimental data recorded by FBG interrogator with a frequency of 200 Hz, from which we can see that the amplitude and frequency of the alternating displacement are 30 mm and 8 Hz, respectively. Table 1 shows the changes of sensitivity and range at different cycle numbers. The sensitivity values have no abnormal change, which demonstrate that the alternating displacement does not degrade the measurement capability.

**Figure 13:**
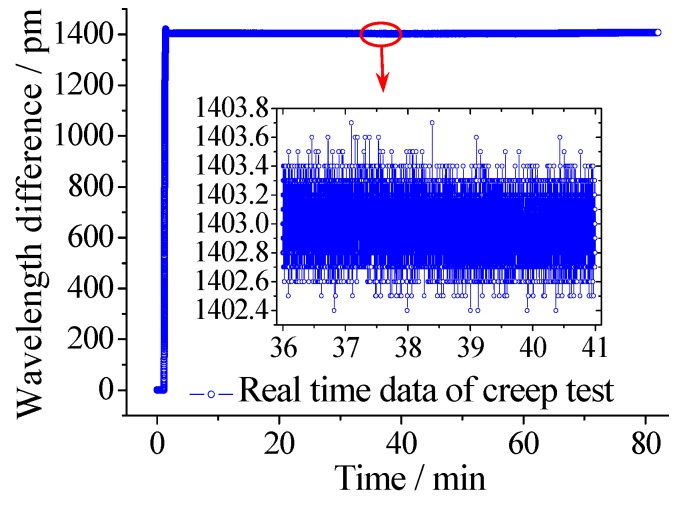
Result of creep performance test for displacement measurement.

Creep performance has a significant influence on the measuring accuracy of sensor for long-term monitoring of floating slab damping track. Therefore, it is necessary to investigate the creep performance of the proposed sensor. During this test, the pull rod was pulled out to a certain position and fixed for more than 80 minutes. The FBG interrogator recorded the wavelength shifts change of the two FBGs. The time-history curve is shown in Figure 13, and the data of 36~41 min is selected and amplified. As we can see, the fluctuating value is within 2 pm, which demonstrates that the sensor has good creep resistance.

## 4. Conclusions

This work proposed and demonstrated the design and performance test of an FBG displacement sensor with high abrasion resistance for a steel spring floating slab damping track. The use of a guide rail-slider system and a precision rolling bearing has been validated to effectively enhance the measurement accuracy and abrasion resistance of sensor. The measurement principle and theoretical model have been derived and experimentally verified. Full performance tests have been carried out in displacement calibration, temperature compensation, creep, and abrasion resistance. Experimental results show that the sensor has a good sensitivity of 34.32 pm/mm, a high resolution of 0.0029 mm over a measuring range of 0~90 mm, a good measurement capability of micro-displacement within a range of 0~3 mm, a repeatability error of 1.416%, and a hysteresis error of 0.323%. The creeping and alternating displacement tests illustrate good creep resistance and excellent abrasion resistance. Compared with the reported FBG displacement sensors, the proposed displacement sensor promises many potential applications in SHM, especially in the displacement monitoring of floating slab damping track that demands for high measurement accuracy and frequent alternating displacement-induced friction.

## Figures and Tables

**Table 1:** Changes of sensitivity under alternating displacement test.

Number of Cycles	Sensitivity (pm/mm)
0	34.32
10^2^	34.61
10^3^	34.21
10^4^	34.66
10^5^	34.71
